# A Recombinant Zika Virus Envelope Protein with Mutations in the Conserved Fusion Loop Leads to Reduced Antibody Cross-Reactivity upon Vaccination

**DOI:** 10.3390/vaccines8040603

**Published:** 2020-10-13

**Authors:** Beatrice Sarah Berneck, Alexandra Rockstroh, Jasmin Fertey, Thomas Grunwald, Sebastian Ulbert

**Affiliations:** Fraunhofer-Institute for Cell Therapy and Immunology, 04103 Leipzig, Germany; beatrice.berneck@izi.fraunhofer.de (B.S.B.); alexandra.rockstroh@izi.fraunhofer.de (A.R.); jasmin.fertey@izi.fraunhofer.de (J.F.); thomas.grunwald@izi.fraunhofer.de (T.G.)

**Keywords:** Zika virus, E-protein, fusion loop, mutations, vaccine, dengue virus

## Abstract

Zika virus (ZIKV) is a zoonotic, human pathogenic, and mosquito-borne flavivirus. Its distribution is rapidly growing worldwide. Several attempts to develop vaccines for ZIKV are currently ongoing. Central to most vaccination approaches against flavivirus infections is the envelope (E) protein, which is the major target of neutralizing antibodies. Insect-cell derived, recombinantly expressed variants of E from the flaviviruses West Nile and Dengue virus have entered clinical trials in humans. Also for ZIKV, these antigens are promising vaccine candidates. Due to the structural similarity of flaviviruses, cross-reactive antibodies are induced by flavivirus antigens and have been linked to the phenomenon of antibody-dependent enhancement of infection (ADE). Especially the highly conserved fusion loop domain (FL) in the E protein is a target of such cross-reactive antibodies. In areas where different flaviviruses co-circulate and heterologous infections cannot be ruled out, this is of concern. To exclude the possibility that recombinant E proteins of ZIKV might induce ADE in infections with related flaviviruses, we performed an immunization study with an insect-cell derived E protein containing four mutations in and near the FL. Our data show that this mutant antigen elicits antibodies with equal neutralizing capacity as the wildtype equivalent. However, it induces much less serological cross-reactivity and does not cause ADE in vitro. These results indicate that mutated variants of the E protein might lead to ZIKV and other flavivirus vaccines with increased safety profiles.

## 1. Introduction

Zika virus (ZIKV) belongs to the Flaviviruses, a genus of small, enveloped RNA viruses, which include several human pathogens, such as Dengue (DENV), West Nile (WNV), Tick-borne Encephalitis, or Yellow Fever viruses [1]. ZIKV is transmitted by mosquitoes and can induce disease symptoms ranging from fever to severe neurological complications including the Guillain-Barré-Syndrome. In addition, ZIKV infections have been linked to birth defects in newborns (e.g., microcephaly) [2,3]. The virus was first identified in 1947 and remained largely unnoticed until its emergence in the South Pacific in 2007 and its following spread over South and Middle America and other parts of the world [4,5,6]. Recently, the first locally acquired cases have been documented in Europe [7].

As with most flaviviruses, an infection with ZIKV leads to long-lasting protection against re-infection. At the same time, due to the structural similarity of flaviviruses, immune cross-reactivity is observed. This might lead to the phenomenon of antibody-dependent enhancement of infection (ADE) [8,9]: antibodies from a previous infection with a different flavivirus bind to, but cannot neutralize the virus, which leads to its increased uptake into host cells via Fc-receptor mediated endocytosis and thereby to increased viremia in the early phase of infection. ADE has been described as a consequence of different DENV-serotypes, but was also seen with DENV and ZIKV antibodies in vitro and in animal models [10,11,12,13].

The RNA genome of ZIKV encodes the structural proteins C, pr/M and E, which form the viral particle, as well as seven non-structural proteins. The envelope protein E is the major component of the virus envelope and responsible for cell attachment and invasion. It is the most important target for protective antibodies in flavivirus infections and therefore a critical component of vaccines. Recombinant forms of the E-protein have been generated in various expression systems. Insect-cell derived variants have entered clinical vaccine-trials for DENV and WNV [14,15]. For ZIKV, immune responses elicited by recombinantly expresses E-proteins, including a *Drosophila S2* cell derived protein, have been studied in detail, and results demonstrate their potential to protect animals from infection [16,17,18,19,20].

The E-protein includes highly conserved parts, most importantly the fusion loop domain (FL), a short amino-acids sequence which is almost identical in many clinically relevant flaviviruses, including ZIKV and DENV [21]. The FL is targeted by cross-reacting antibodies and has been linked to ADE development [22,23]. This represents a challenge for vaccine development, as vaccine-induced immunity against one flavivirus could lead to enhancement of infection with a different one. Especially in areas of co-circulation of multiple flaviviruses, this is of concern.

Therefore, we have studied a mutant form of the E protein (termed Equad), containing four points mutations in and near the FL as an alternative vaccine candidate. The protein with all four mutations has previously been shown to greatly reduce the binding of cross-reactive antibodies from other flavivirus infections and therefore to be useful in diagnostic serology [24]. We show here that the same recombinant Equad protein elicits immune responses comparable to the wildtype protein but induces significantly fewer cross-reactive antibodies and no ADE.

## 2. Materials and Methods

### 2.1. Cells and Viruses

*Drosophila S2* cells (Invitrogen, Carlsbad, CA, USA) were propagated in Schneider’s medium supplemented with 10% FCS and 1% penicillin/streptomycin at 28 °C. Vero E6 (DSMZ, Braunschweig, Germany) cells were maintained in Dulbecco’s modified Eagle’s medium (DMEM) supplemented with 10% FCS and 1% penicillin/streptomycin at 37 °C with 5% CO_2_. K562 cells were cultivated in Roswell Park Memorial Institute (RPMI) 1640 medium added with 10% FCS, 2 mM Glutamine and 1% penicillin/streptomycin at 37 °C with 5% CO_2_. All viruses were grown in Vero E6 cells. ZIKV (Padova; Dominican Republic/2016/PD1; GenBank KU853012, kindly provided by Luisa Barzon, Padova University, Padova, Italy) was purified as previously described [25]. After ultracentrifugation, viral pellets were resuspended in PBS containing 10% (w/v) sucrose. The purified virus was stored in aliquots at −80 °C until use. DENV-1 to -4 (DENV-1 isolate 2522/10, DENV-2 isolate 3229/11, DENV-3 isolate 3140/09, DENV-4 isolate 3274/0, kindly provided by Jonas Schmidt-Chanasit (Bernhard Nocht Institute for Tropical Medicine, Hamburg, Germany) were purified from virus containing cell culture supernatants 2–6 days post infection (dpi) by ultracentrifugation for 3 h at 4 °C. Viral pellets were resuspended in PBS with 10% (w/v) sucrose and stored in aliquots at −80 °C until use.

ZIKV titers were determined via the tissue culture infectious dose 50 (TCID50) assay based on endpoint dilutions. Briefly, 10-fold dilutions of viral stocks were incubated on Vero E6 cell monolayers and the cytopathic effect (CPE) was monitored 5 dpi. The 50% infectious dose was calculated using the Reed–Muench method [26].

DENV-1 to -4 titers were determined by a focus forming assay. Viruses were serially diluted on Vero E6 cell monolayers in 96-well plates. After 1 h, the supernatant was removed and cells were overlaid with 1% methylcellulose in DMEM with 2% FCS and 1% penicillin/streptomycin. Two days later, the cells were fixed with 1% formaldehyde in PBS, permeabilized and blocked with Perm-Wash buffer (0.1% Saponin and 0.1% BSA in PBS). After the incubation of the primary flavivirus antibody 4G2 (Absolute antibody, Oxford, UK, 1:2000), several wash steps were applied with Perm-Wash buffer and followed by the incubation with an anti-mouse IgG HRP-conjugated secondary antibody (Dako, Glostrup, Denmark, 1:2000). For the detection of focus forming units, TrueBlue peroxidase substrate (Seracare Life Sciences, Milford, MA, USA) was used and the spots were counted automatically with an ELISpot reader (AID Diagnostika, Straßberg, Germany).

For ELISA measurements, ZIKV and DENV-1 to -4 were inactivated by treatment with 0.3% H_2_O_2_ in PBS at 37 °C overnight. Subsequently, the samples were dialyzed against PBS and stored in aliquots at −80 °C until further use.

### 2.2. Expression and Purification of Recombinant Proteins

The ectodomain of the ZIKV E-protein (strain H/PF/2013, E-protein amino acid residues 1–406) bearing four mutations, T76A, Q77G, W101R and L107R, termed quadruple mutant or Equad, was expressed in stably transfected *Drosophila S2* cells and purified from cell culture supernatants with immobilized imidazole affinity and size exclusion chromatography as previously described [24]. The E wildtype protein (Ewt) was generated accordingly. The E-protein ectodomains of forms of DENV and WNV have been described previously [27,28]. All constructs were verified by DNA sequencing before protein expression.

### 2.3. Animal Experiments

All animal experiments were carried out in accordance with the EU Directive 2010/63/EU for animal experiments and were approved by local authorities (No.: TVA 02/18).

Groups of 6 (*n* = 6) nine weeks old, female BALB/c mice were immunized three times in 4-week intervals by intramuscular injection (hind leg) with 15 µg of either ZIKV Equad or ZIKV Ewt with the adjuvant 2% Alhydrogel (Invivogen, San Diego, CA, USA) at a ratio 1:1 (*v/v*). Control mice were not vaccinated. Serum samples were collected from blood before the first injection (prime) and thereafter before the boost immunizations by retrobulbar bleeding. Final blood sampling was performed 4 weeks after the second boost. The samples were inactivated for 30 min at 56 °C and stored in aliquots at −20 °C until use.

### 2.4. ELISA Analysis

Mouse sera were tested on microtiter plates coated either with the recombinant proteins ZIKV Equad, ZIKV Ewt, DENV Ewt (respectively 150 ng per well), WNV Ewt (200 ng per well) or inactivated viral particles in 100 µL buffer (15 mM Na_2_CO_3_, 7 mM NaHCO_3_ pH 9.6) overnight at 4 °C. The plates were then washed three times with PBS-0.05% Tween and blocked with 5% non-fat milk powder in PBS for 2 h at room temperature (RT). After another wash step, the incubation started with diluted mouse sera for 1.5 h at RT. Following a third wash step, the HRP-conjugated secondary anti mouse IgG antibody (Dako, Glostrup, Denmark, 1:3000) was added for 1 h at RT. After a final wash step, TMB substrate (Biozol, Eching, Germany) was added and incubated for 30 min at RT, before the reaction was stopped with 1 M H_2_SO_4_. The plates were analyzed at 450 nm with 520 nm as reference in a microplate reader (Infinite M200, Tecan, Männedorf, Switzerland). The ELISA assays on ZIKV proteins were performed with 1:1000 diluted sera, on DENV and WNV proteins serial diluted sera were used to determine endpoint titers. These were defined as the reciprocal of the highest dilution with signals were above the cut-off based on the control group which were calculated as the mean plus 8 standard deviations (SD) of sera from the control group. For the ELISA assay on inactivated viral particles, sera were diluted 1:100. Human sera were diluted 1:100 for ELISA assays on ZIKV Equad and Ewt and the secondary HRP-conjugated antibody anti human IgG was diluted 1:20,000 (Dianova, Hamburg, Germany). The human sera were kindly provided by Luisa Barzon (Padova University, Italy) and Jonas Schmidt-Chanasit (Bernhard Nocht Institute for Tropical Medicine, Hamburg, Germany). Ethical approvals were obtained from the respective local authorities for all samples. The 4G2 antibody (Absolute antibody, Oxford, UK) was diluted 1:1000 and the secondary mouse-antibody (Dako, Glostrup, Denmark) was diluted 1:3000. Tests were performed in duplicates and in two independent experiments.

### 2.5. Virus Neutralization Assay

Serially diluted mouse sera taken 4 weeks after second boost were incubated with infectious supernatant of ZIKV at multiplicity of infection of 0.0015 for 1 h at 37 °C and incubated on a Vero E6 cell monolayer in microtiter-plates. After 3 h, the medium was changed. The CPE was determined 5 dpi and cells were fixed with 70% ethanol and stained by crystal-violet solution. Serum titer was defined as the reciprocal of the highest dilution showing no CPE.

### 2.6. ADE Assay

Heat inactivated mice sera were diluted 1:320 (based on initial titration, data not shown) and incubated with DENV-2 (isolate 3229/11) with a multiplicity of infection of 0.1 for 1 h at 37 °C. Then, these mixtures were added to K952 cells and incubated for 2 h. A wash step was applied by adding RPMI 1640 medium with 10% FCS, 2 mM Glutamine and 1% penicillin/streptomycin and subsequent centrifugation. After 2 days, the cells were analyzed by flow cytometry (FACSCanto II, Becton Dickinson, Franklin Lakes, NJ, USA) using the flavivirus-specific 4G2 antibody (1:500) and a FITC-conjugated anti mouse antibody for detection (Dako, Glostrup, Denmark, 1:50). The n-fold increase of enhancement was calculated as: (numbers of positive cells/numbers of all cells in the presence of immunized mouse sera)/(numbers of positive cells/numbers of all cells in the presence of control mouse sera) [29]. Tests were performed in two independent experiments.

### 2.7. Statistics

Statistical analysis was performed using GraphPad Prism6 (GraphPad Software, Inc., La Jolla, CA, USA). Statistical tests were calculated as ordinary one-way unpaired ANOVA or unpaired *t*-test, with * = *p* < 0.05, ** = *p* < 0.01, *** = *p* < 0.001, **** = *p* < 0.0001, ns = not significant.

## 3. Results

**Figure 1:**
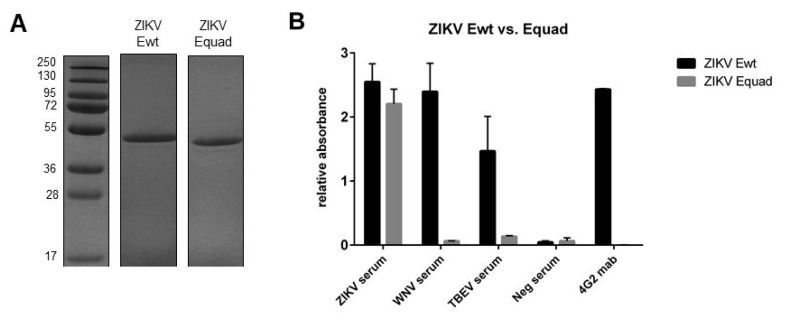
Analysis of recombinantly expressed ZIKV Equad and Ewt. (**A**) Proteins after imidazole affinity purification and size exclusion chromatography on a 12% SDS polyacrylamide gel stained with Coomassie blue; (**B**) Comparison of cross-reactivity between ZIKV Equad and Ewt using an IgG-ELISA with human sera positive for ZIKV, WNV or TBEV and the FL-specific monoclonal 4G2 antibody. A flavivirus negative serum served as control.

We investigated an FL mutated ZIKV E protein, bearing four point mutations (Equad) on its potential to reduce cross reactivity with other flaviviruses and ADE after vaccination in comparison to the corresponding wildtype protein (Ewt). Both proteins were expressed in *Drosophila S2* cells and purified via two chromatography steps (Figure 1A). To verify that the FL domain was indeed mutated in the Equad protein, a serological analysis was carried out. In an ELISA, serum from a ZIKV-infected human recognized both Ewt and Equad, but sera from humans infected with WNV and TBEV only bound Ewt (Figure 1B), confirming previous studies [24]. In addition, an FL-specific monoclonal antibody only bound Ewt but not Equad (Figure 1B). These results indicate that both proteins are identical with the exception of the FL structure, which is disrupted in Equad.

**Figure 2:**
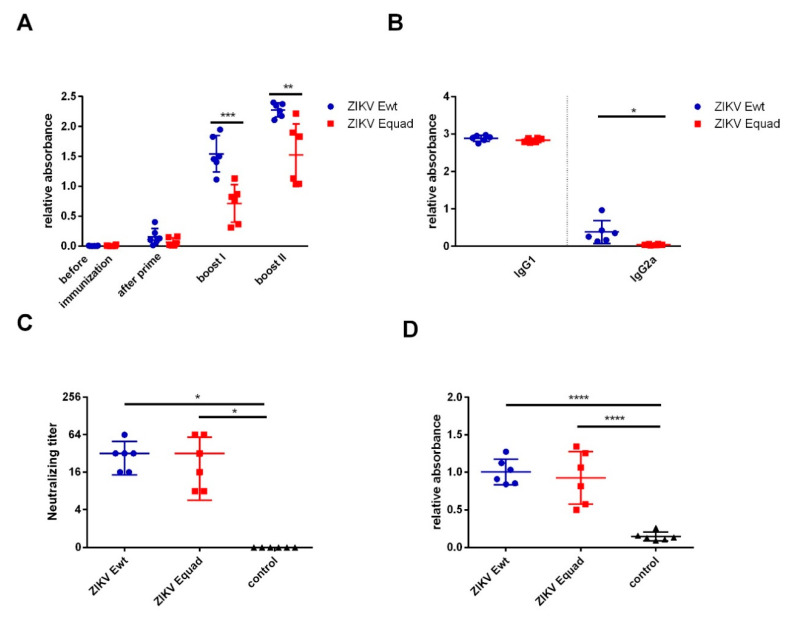
Analysis of humoral immune responses after vaccination with ZIKV Equad and Ewt. (**A**) Serum IgG antibodies from different time points were analyzed by ELISA on the homologous proteins used for the immunizations. (**B**) Determination of IgG subclasses of antibodies after the second boost. (**C**) Neutralizing antibody titers were determined with serum collected 4 weeks after the second boost. (**D**) Sera taken after the second boost were analyzed by ELISA coated with inactivated ZIKV. Data are shown for individual mice as well as mean ± SD for one group. Statistical analysis was performed by a one-way unpaired ANOVA test except for **A** and **D** where an unpaired *t*-test was used (* = *p* < 0.05, ** = *p* < 0.01, *** = *p* < 0.001, **** = *p* < 0.0001).

Mice were immunized three times with either ZIKV Ewt or Equad recombinant proteins expressed in *Drosophila S2* cells with aluminium hydroxide as adjuvant. The animals were tested for the development of a specific IgG response by ELISAs using the respective antigen. After the first boost, both groups mounted a response, and the ZIKV Ewt group showed significantly higher signals compared to the ZIKV Equad group. Following the second boost, the signals further increased in both groups (Figure 2A). The antibody isotypes were analyzed by ELISA on the homologous proteins. In both groups IgG1 was the dominant isotype, with only very little IgG2 detectable (Figure 2B). To determine the neutralizing activity of the antibodies, virus neutralization tests (VNT) were performed. ZIKV Ewt as well as the Equad groups elicited neutralizing titers at an average of 1:32 that did not differ significantly (Figure 2C). In addition, sera from the second boost were analyzed on whole inactivated ZIKV particles, where equal binding was observed in both groups (Figure 2D).

**Figure 3:**
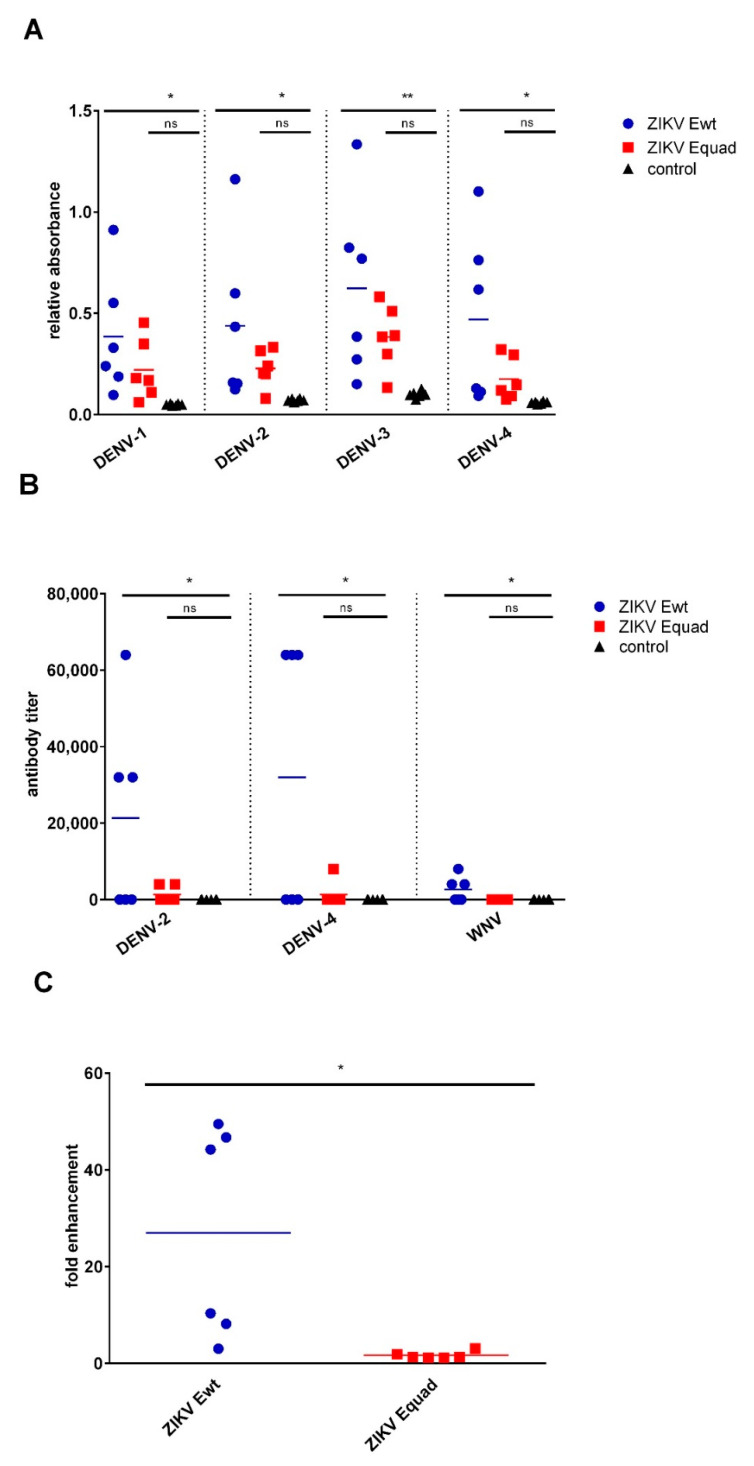
Binding properties of induced antibodies against heterologous flaviviruses. (**A**) Analysis of mouse sera for binding to inactivated DENV-1 to -4; (**B**) Antibody titers of mouse sera against Ewt proteins of DENV-2, DENV-4 and WNV. (**C**) ADE assay with immunized mouse sera after incubation with DENV-2. The infection of K562 cells was analyzed. Mouse sera after the second boost were used for these experiments. Data are shown for individual mice as well as mean ± SD for one group. Statistical analysis was calculated by ordinary one-way unpaired ANOVA test except for **C** where an unpaired *t*-test was used (* = *p* < 0.05, ** = *p* < 0.01, ns = not significant).

To investigate the potential cross-reactivity, serum antibodies were tested for binding to whole inactivated DENV-1 to -4 particles and against recombinant E wildtype proteins of DENV-2, DENV-4 and WNV. The group immunized with Ewt showed significantly higher ELISA signals on DENV-1 to -4 as compared to the ZIKV Equad immunized group. The amount of binding antibodies in the Equad immunized sera was higher than those of the control animals but the difference was not statistically significant (Figure 3A). Likewise, the antibody titers of the mouse sera against recombinant Ewt proteins of heterologous flaviviruses were significantly higher in the ZIKV Ewt immunized group as compared to the Equad immunized group, which was similar to the control animals (Figure 3B). The binding to heterologous flavivirus and E wildtype protein was mainly attributed to three mice within the Ewt-immunized group, hence sera from half of the animals displayed cross-reactive binding, the other half did not.

Subsequently, the potential antibody-dependent enhancement (ADE) of DENV infection was investigated. To this end, DENV-2 was mixed with diluted mouse sera and then used to infect K952 cells. After 2 days, the cells staining positive with a DENV antibody were counted. ADE activity was absent for the ZIKV Equad immunized group, similar to the negative control samples. In contrast, in the Ewt immunized group, DENV infection due to ADE was strongly induced by three sera, and modest enhancement was seen with two sera (Figure 3C). The three sera displaying the highest enhancements were the ones with the highest cross-reactivity towards the heterologous proteins (Figure 3B).

## 4. Discussion

The E-protein of flaviviruses, including ZIKV, serves as the main target of neutralizing antibodies and is central to many different vaccination approaches [30]. However, cross-reacting antibodies that mainly target the conserved FL domain of the E-protein could potentially enhance infections with heterologous flaviviruses. Because of the overall increase in flavivirus spread and growing areas of co-circulation of different flaviviruses this needs to be addressed during vaccine development. The aim of the present study was to analyze the effect of disrupting the FL-structure of a *Drosophila S2* cell derived E-protein, as this antigen represents a very advanced candidate for ZIKV vaccines and has already entered clinical trials for other flavivirus vaccines.

We show that the inclusion of four amino acid replacements in and near the FL does not interfere with the general functionality of the E-protein as vaccine antigen. This protein induced IgG responses which were detectable after the first boost and strongly increased after the second. The lower signals of IgG to the homologous antigen elicited by Equad compared to Ewt could be explained by the lack of the FL-binding antibodies, which lead to increased signals in the Ewt-immunized group. On the inactivated ZIKV particles, where the FL epitope is less exposed [31], the difference between both groups was much smaller. Both proteins induced a Th2-type response, as concluded from the predominant IgG1 isotype in the animal sera. This is likely due to the adjuvant used, aluminium hydroxide, which is known to be Th2-prone. By using different adjuvants, the Th1-Th2 balance elicited by an E-protein vaccine can be influenced [32]. Most importantly, the Equad protein induced the same amounts of ZIKV-neutralizing antibodies as the Ewt variant.

Although both proteins behaved similarly in the induction of immune responses against the homologous virus (ZIKV), cross-reactive responses to heterologous flaviviruses showed significant differences. Whereas the binding of antibodies to DENV or WNV derived antigens was low in the Equad group, half of the animals immunized with Ewt displayed strong cross-reactivity to both recombinant and native antigens of these viruses, confirming previous observations [19,33]. Moreover, sera from the Ewt immunized, but not from the Equad immunized mice led to the enhancement of DENV infections in an in vitro ADE assay. It is interesting that three animals in the Ewt group showed higher cross-reactivity than the remaining three, although the ZIKV-specific responses were very similar within this group. This indicates that the degree of flavivirus antibody cross-reactivity is not directly linked to the immune response against the homologous virus. To get more insight into the degree of variation in cross-reactive immune responses in Ewt immunized animals, larger experimental groups might be immunized in future studies.

ADE between ZIKV- and DENV-immune sera has been described in several studies [34,35,36], and could represent a severe problem for ZIKV or DENV vaccines. Most ZIKV vaccine approaches based on the E-protein include the FL and do not address this issue, but it has been considered in the development of novel vaccine platform technologies. By using a mRNA-based vaccine encoding the prM and E proteins with the same point mutations in E as in the present study, Richner et al. demonstrated ADE reduction with sera from immunized mice [37]. Artificial E-dimers expressed in mammalian HEK293T cells which reduce the exposure of FL also displayed greatly reduced cross-reactivity and ADE as compared to a wildtype E-monomer [38]. Other approaches only use domain 3 of the E-protein, which excludes the FL and other large proportions of the protein [39,40].

Our results add up to these studies, but by using a more state-of-the-art approach. *Drosophila S2* cells are a well-established production method for flavivirus E proteins in clinical development, with proven safety records. By replacing existing wildtype-based E-protein vaccine candidates for ZIKV with Equad sequences, the potential problem of ADE induction could be met with no consequences for the protective immunity. Moreover, as Equad proteins for other flaviviruses than ZIKV also exist and have been shown to be much less cross reactive than their wildtype counterparts, the technology might also be used for other flavivirus vaccines [27,41].

## 5. Conclusions

In summary, our data suggest that the structural disruption of the highly conserved FL in the insect cell derived ZIKV E protein has negligible consequences for the immunogenicity against ZIKV. However, it induces much less cross-reactive antibodies against other flaviviruses and is therefore a promising alternative to produce ZIKV vaccines with increased safety profiles.
